# Enhanced High-Temperature DC Dielectric Performance of Crosslinked Polyethylene with a Polystyrene Pinning Structure

**DOI:** 10.3390/ma12081234

**Published:** 2019-04-15

**Authors:** Liang Cao, Lisheng Zhong, Yinge Li, Kai Zhang, Jinghui Gao, George Chen

**Affiliations:** 1 State Key Laboratory of Electrical Insulation and Power Equipment, Xi’an Jiaotong University, Xi’an 710049, China; cl.09041187@stu.xjtu.edu.cn (L.C.); ge_chrissy@163.com (Y.L.); zhangkai.925@stu.xjtu.edu.cn (K.Z.); gc@ecs.soton.ac.uk (G.C.); 2 School of Electronics and Computer Science, University of Southampton, Southampton SO16 1BJ, UK

**Keywords:** DC dielectric performance, crosslinked polyethylene, polystyrene, pinning structure

## Abstract

In this paper, we propose a method on improving direct current (DC) dielectric performance by designing a polystyrene (PS) pinning crosslinked polyethylene (XLPE) for the application of insulation materials on high voltage direct current (HVDC) extruded cable. Electrical experimental results show that the addition of PS (1–5 phr, parts per hundreds of resin) can significantly reduce DC conductivity and increase DC breakdown strength of XLPE in the test temperature range of 30–90 °C. Microstructure investigation shows PS distributed as particles could participate in the formation of a crosslinking network with the help of a crosslinking agent, thus forming a polymer pinning structure at the interface between XLPE and PS. It is believed that such a special design strengthens the structure of XLPE, which leads to the improved DC dielectric performance at elevated temperatures. Our findings may contribute a new solution for developing HVDC cable insulation materials.

## 1. Introduction

Crosslinked polyethylene (XLPE) has been employed as the mainstream insulation material for high voltage direct current (HVDC) extruded cables owing to its excellent comprehensive performance and maturity in cable industry utilization. Nowadays the increasing voltage and capacity for HVDC projects triggers a crucial requirement for the HVDC power cable system, in particular that the cable insulation materials work in a higher electric field and thermal conditions [1,2,3,4]. In order to fulfill the specific demand for HVDC application, tremendous efforts have been devoted to modifying the present XLPE insulation materials or the primary resin low density polyethylene (LDPE) in multiple ways. For non-filled XLPE, unnecessary deterioration of direct current (DC) performance in high electric fields has been avoided through restricting the impurities caused by base materials and crosslinking reactions (e.g., employing purified LDPE and lowering the crosslinking degree) [5,6,7]. In nano-composites, polymers filled with appropriate nano-particles have been found to exhibit the superior properties of reduced DC conductivity and enhanced DC breakdown strength, originating from the suppression of space charges caused by nano-particle/polymer interfaces [8,9,10]. Blending and grafting have also been employed to modify DC dielectric performance and manipulate the space charge behavior for XLPE or non-crosslinking polyolefin [11,12,13,14,15]. It should be noticed that although the above-mentioned methods succeed in improving the DC performance of polyethylene, the cable insulation materials have always been subjected to stringent conditions with high electric fields at an elevated temperature, which largely changes DC dielectric properties and thus leads to a high degree of electric field distortion triggering possible breakdown. Therefore, it is of great importance to develop cable insulation materials which exhibit better DC dielectric properties at high temperatures in order to fulfill the enhancing criteria for high performance HVDC extruded cable insulation materials. 

In this paper, we propose a strategy that can enhance the DC properties of XLPE through deliberately introducing homogeneously-dispersed polystyrene (PS) particles. This method effectively increases the breakdown strength and lowers the DC conductivity at elevated temperatures up to 90 °C compared with the conventional XLPE, which indicates its possible application as a promising cable insulation material.

## 2. Materials and Methods

### 2.1. Sample Preparation

A commercial LDPE was used as the primary resin with a melt index of 2.0 ± 0.2 g/10 min (190 °C, 2.16 kg) and a density of 0.923 g/cm^3^. A PS was used with a melt index of 0.32 g/10 min (190 °C, 2.16 kg), a density of 1.05 g/cm^3^, and heat deflection temperature of 98 °C. The antioxidant 300 was employed and dicumyl peroxide (DCP) was selected as the crosslinking agent.

First, pellets of LDPE and PS and antioxidant were carefully weighed using a digital balance. After pre-mixing, pellets and antioxidant were blended by a twin screw extruder at 170 °C with a speed of 60 r/min, through which PS was dispersed and distributed in LDPE. Then a certain amount of DCP was add to pellets of LDPE with PS by soaking at 70 °C for 24 h [16]. Plate samples with different thicknesses were prepared by hot pressing with a vulcanizer. Pellets with DCP were made into plate samples at 120 °C /15 MPa for 5 min, then at 180 °C /15 MPa for 15 min and cooled to room temperature. Different contents of PS including 1 phr, 3 phr, and 5 phr (phr: parts per hundreds of resin) were introduced into LDPE and XLPE samples with PS and were marked as XLPE-1PS, XLPE-3PS, and XLPE-5PS respectively. Before tests, all the samples were pre-treated at 70 °C for 24 h to remove by-products and relax the internal stress.

### 2.2. DC Dielectric Performance Measurements

A three-electrode system was used in the DC conductivity measurements and the diameter of the measuring electrode was 25 mm. The whole electrode system was put in an oven, for which the test temperature was set to 30 °C, 50 °C, 70 °C, and 90 °C, respectively. A DC electric field of 20 kV/mm was applied to the samples and the current was measured by a Keithley (Beaverton, OR, USA) 6517B and recorded for 60 min by a computer [17], the median of the last 1 min current was taken as the current for each sample. For each recipe, the average conductivity of three samples were calculated. 

The pulsed electro-acoustic (PEA) method was used to analyze space charge characteristic of all the samples. The anode electrode (top) was a semiconducting electrode while the cathode was aluminum. Different electric fields including 20 kV/mm and 50 kV/mm were applied to all the samples, and space charge measurements lasted for 60 min.

DC breakdown tests were performed with 25 mm cylindrical electrodes. Plate samples, electrodes and a thermometer were immersed in a vegetable transformer oil in order to provide a stable environment and avoid flashover in air, as used in our previous study [18]. Test temperatures were set to 30 °C, 50 °C, 70 °C, and 90 °C respectively, and the rate of DC ramp voltage was approximately 1 kV/s. The results from 20 tests for each recipe were analyzed by two-parameter Weibull distribution with 95% confidence intervals for Weibull parameters.

### 2.3. Morphology and Structure Characterization

The crosslinking degree of all the samples were evaluated by gel content. Each sample was cut into small cubes with a dimension of approximately 0.5 mm × 0.5 mm × 0.5 mm and then placed in a 120 mesh stainless steel net bag. The whole bag was extracted with xylene as the solvent at 110 °C for 24 h. After extraction, the samples were dried to a constant weight at 150 °C. Gel content was calculated as the percentage ratio of the final weight of the polymer to its initial weight. Further, gels coming from XLPE extracted by xylene were analyzed by a Nicolet IN10 spectrometer recorded from 400 cm^−1^ to 4000 cm^−1^. The resolution was 4 cm^−1^ with a total of 32 scans.

Etched surface observation and X-ray diffraction (XRD, Bruker, Karlsruhe, Germany) analysis of samples were employed to analyze crystal structure and morphology. Samples with a thickness of ~250 μm were immersed in a 5 wt% solution of potassium permanganate in concentrated sulfuric acid at 30 °C for 4 h. Then etched samples were washed with dilute sulfuric acid, hydrogen peroxide, distilled water, and acetone in sequence, during which the ultrasonic vibration was applied. After drying in air, the samples were coated with gold and observed by a scanning electron microscope (SEM, Keyence, Osaka, Japan). The XRD was performed on a Bruker (Karlsruhe, Germany) D8 advance A25 diffractometer operating at 40 kV and 40 mA. The data was scanned from 10° to 60° at a rate of 2°/min. The crystallinity of samples were calculated by peak area analysis. 

Furthermore, SEM was used to observe cryogenically fractured cross sections of all the materials separately. Samples were immersed in liquid nitrogen for 3 min and then fractured. After coating with a thin layer of gold, fracture surfaces were observed by SEM.

## 3. Results

### 3.1. DC Conductivity Properties

For an insulation material in DC power cable application, low conductivity, minimized space charge accumulation, and high breakdown strength are highlighted. Therefore these DC dielectric properties are demonstrated.

**Figure 1:**
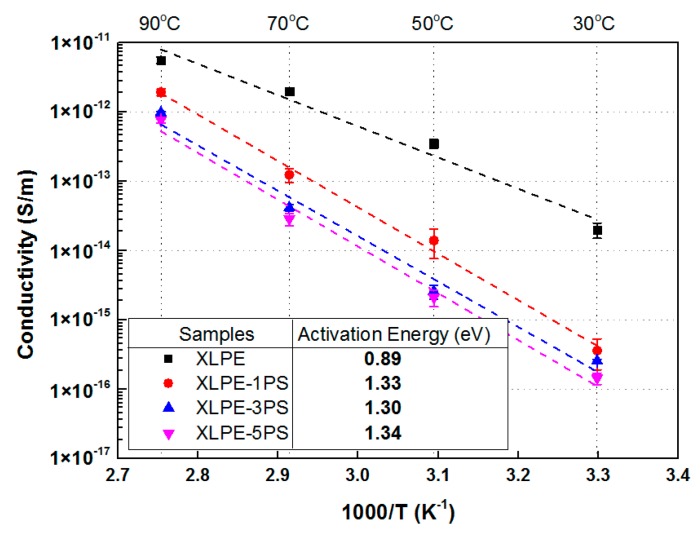
Temperature dependence of DC conductivity under 20 kV/mm for samples.

Figure 1 shows the DC conductivity of samples under 20 kV/mm from 30 °C to 90 °C. The results indicate that DC conductivity of samples increases with the rise of temperature. DC conductivity of materials with PS were much lower than that of materials without PS in the test temperature range. At 70 °C, there was a scalar difference in conductivity between XLPE and XLPE with PS. At 90 °C, the conductivity of XLPE was several times larger than that of XLPE with PS. 

Under the electric field of 20 kV/mm, the conduction in polymer may be a hopping mechanism and charge carriers are transferred from localized sites (traps) to another by thermal activation. The thermal activation energy could be obtained from an Arrhenius plot [19]. The increase of slopes for fitting curves represents the increase of activation energy and it could be calculated that the addition of PS into XLPE increased the activation energy from 0.89 eV (XLPE) to above 1.3 eV (XLPE-1PS: 1.33 eV, XLPE-3PS: 1.30 eV, and XLPE-5PS: 1.34 eV). The addition of PS increased the difficulty of carriers hopping, and thus reduced DC conductivity in the test temperature range.

### 3.2. Space Charge Behaviors

**Figure 2:**
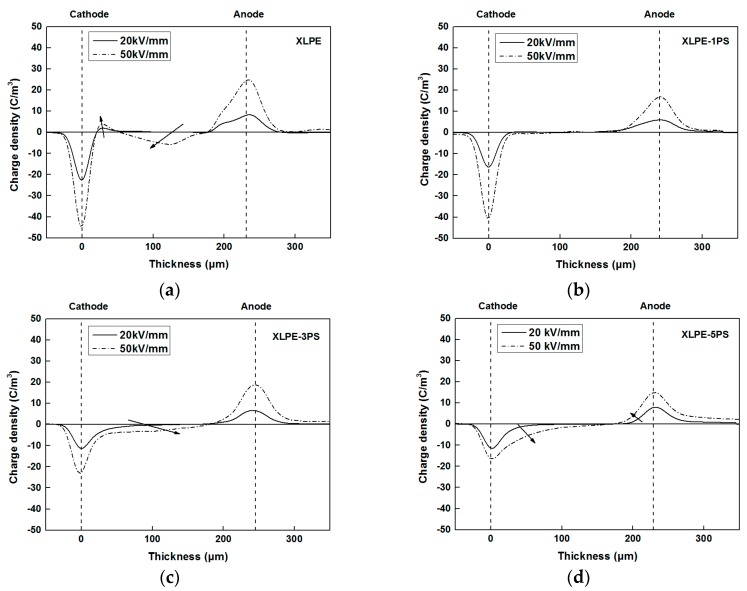
Space charge distribution of samples of (**a**) crosslinked polyethylene (XLPE); (**b**) XLPE-1PS; (**c**) XLPE-3PS; (**d**) XLPE-5PS.

Figure 2 presents the space charge distribution of all the samples under different applied electric fields. In the bulk of XLPE, a positive charge near the cathode and a negative charge near the anode indicated the formation of a heterocharge and the charge peak increased with the applied electric field. While in XLPE with PS, apparent homocharge accumulated near the electrodes. With the increase of PS content, charge density of the cathode and anode tended to decrease, indicating that electrode injection was enhanced by the introduce of PS. These results suggest that certain quantities of PS could help to suppress space charge accumulation in XLPE.

### 3.3. DC Breakdown Strength

**Figure 3:**
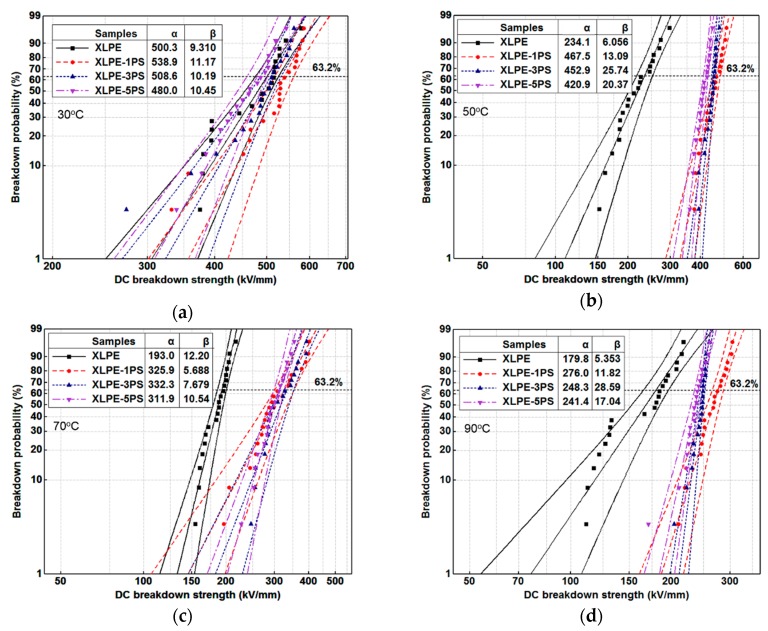
Weibull distribution of DC breakdown strength for samples under different temperatures, (**a**) 30 °C; (**b**) 50 °C; (**c**) 70 °C; (**d**) 90 °C.

Figure 3 shows the Weibull distribution of DC breakdown strength for each recipe from 30 °C to 90 °C respectively. Scale parameter α represents the Weibull statistical breakdown strength, the DC breakdown strength at the cumulative probability of 63.2%, in kV/mm. Shape parameter β shows the data dispersivity. Revealed by β, the fluctuation of the plots in Weibull distribution is related to the microstructure of samples, impurities, crosslinking byproducts, and void. The fluctuation is common in semi-crystalline polymers. Obviously for all the materials, DC breakdown strength declined with the temperature increase. The increase of temperature can cause the decrease of elastic modulus and lead to the melting of crystallites, creating a new amorphous area and enlarging the free volume of XLPE. These phenomena may be the main reason for the reduction of DC breakdown strength with the increase of temperature [5]. However, it could be found that addition of PS into XLPE could enhance DC breakdown strength.

**Figure 4:**
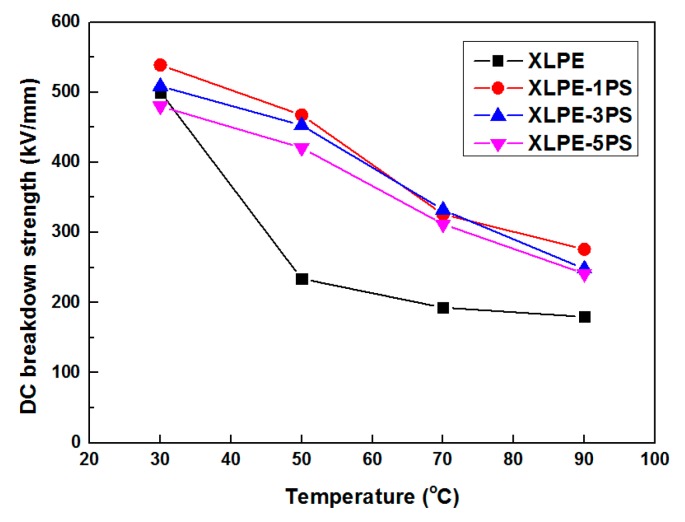
Temperature dependence of DC breakdown strength for samples.

The temperature dependence of DC breakdown strength for material samples are shown in Figure 4, where DC breakdown strength of all the samples, especially for 70 °C and 90 °C, are compared. At 70 °C and 90 °C, DC breakdown strength of XLPE with PS were increased by at least 61.6% and 34.3% respectively. In general, the addition of PS into XLPE decreased the effect of temperature on the DC breakdown strength in the range of test temperature.

### 3.4. Structure Characteristics

In order to explore the mechanism of the enhancement of DC dielectric performance for XLPE by the addition of PS, the microstructure of XLPE with PS, including the crosslinking network, crystal structure, and interface between XLPE and PS, are characterized. 

Results of the gel content experiment show that the crosslinking degree does not exhibit a significant difference. The gel content of XLPE, XLPE-1PS, XLPE-3PS, and XLPE-5PS were 81.68 ± 0.32%, 81.00 ± 0.24%, 80.20 ± 0.02%, and 82.08 ± 0.05% respectively. For XLPE the crosslinking behavior mainly depends on LDPE and DCP, and in these samples, the ratio of LDPE and DCP remained a constant. However, they did show a slight difference in gel content. It is speculated that addition of PS may affect the crosslinking process.

**Figure 5:**
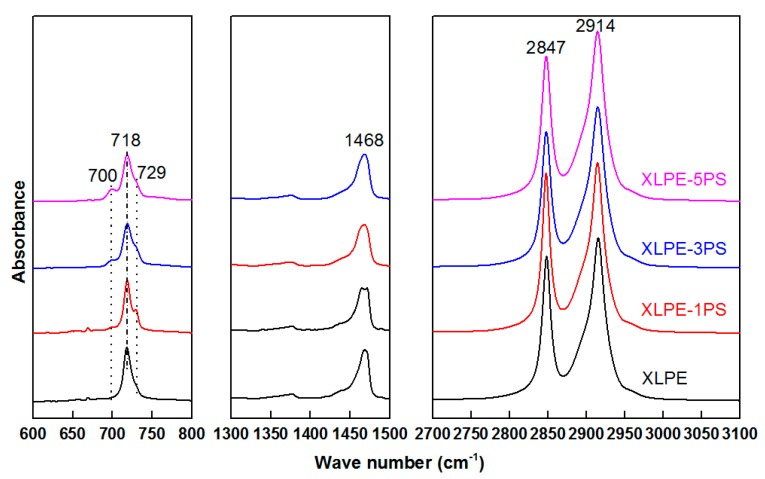
FTIR spectra of the gel for samples.

Further Fourier transform infrared spectroscopy (FTIR) was used to investigate the functional group remaining in the gel content of material samples, namely the network of XLPE. As shown in Figure 5, it was clearly observed that peaks were dominantly belonging to polyethylene. The peaks at 718 cm^−1^ and 729 cm^−1^ represent CH_2_ rocking vibrations in an amorphous area and crystalline area respectively. Peaks at 1468 cm^−1^ represent CH_2_ scissoring bond vibrations. Peaks at 2847 cm^−1^ and 2914 cm^−1^ represent symmetric stretching vibrations and asymmetric stretching vibrations of CH_2_ respectively. Particularly, peaks at 700 cm^−1^, which represent the vibration absorption of CH bound on mono-substituted benzene rings, appear in XLPE-1PS, XLPE-3PS, and XLPE-5PS but not in XLPE, and that is to say, the crosslinking network contains a benzene ring. The most possible reason is that PS was grafted onto XLPE with the help of DCP. Meanwhile, with the increase of PS content, peaks at 700 cm^−1^ also tended to increase. Peaks at 729 cm^−1^ revealed that in the gel of XLPE-1PS there exist molecules that could crystallize. It could be inferred that more DCP was reacted between XLPE and PS, leading to more molecule chains of XLPE that could take part in crystallization. 

**Figure 6:**
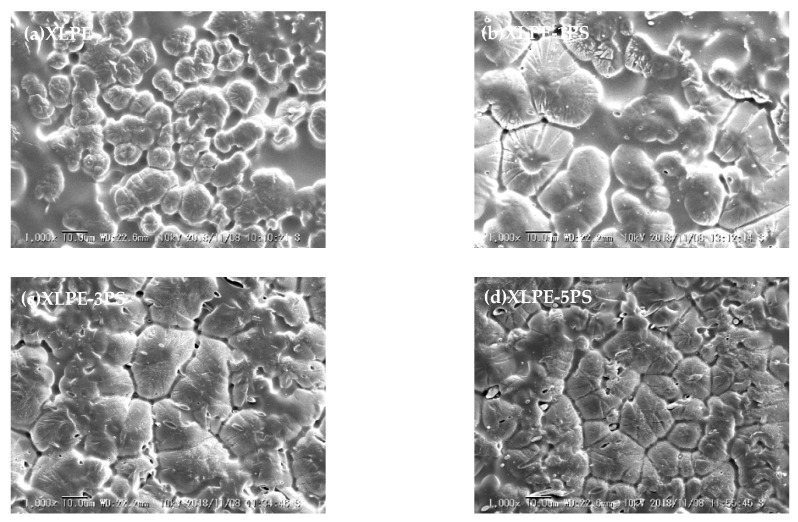
The etched surface morphology of samples by SEM. (**a**) XLPE, (**b**) XLPE-1PS, (**c**) XLPE-3PS, (**d**) XLPE-5PS.

**Figure 7:**
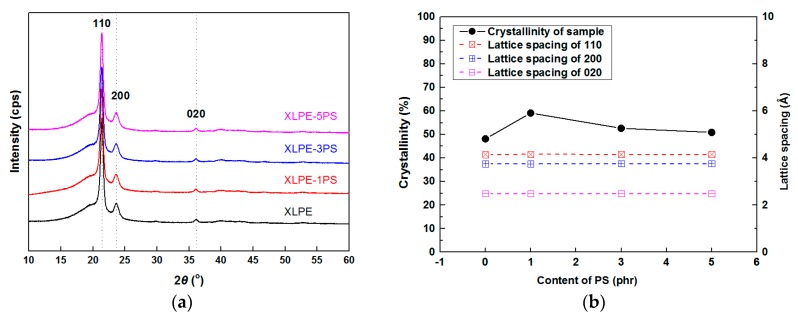
The XRD results of samples. (**a**) XRD spectra; (**b**) crystallinity and lattice spacing calculated from XRD data.

Figure 6a–d shows SEM images of etched surface of samples. The size of spherulites was analyzed, and the average diameter of XLPE, XLPE-1PS, XLPE-3PS, and XLPE-5PS was 8.5 μm, 15.4 μm, 13.4 μm, and 11.4 μm respectively. It was seen that the addition of PS enlarged the crystal size of XLPE, and with the increase of PS content, crystal size of XLPE decreased but tended to become tighter and more uniform. The results of the XRD are presented in Figure 7. 2*θ* of the diffraction peaks in spectra were mainly at 21.4°, 23.7°, and 36.1°, which respectively related to the 110, 200, and 020 lattice planes of polyethylene. 2*θ* of a broad peak originating from the amorphous area of polyethylene appeared at 19.8°. No other peaks could be found, which indicates that addition of PS into polyethylene does not change its crystalline form. *d* spacings were calculated according to the Bragg equation, and there was no significant change for (110), (200), and (020), which remained nearly constant under different contents of PS, which implies that the lamellar structure of XLPE does not vary with the content of PS.

The crystallinities could be calculated from the following equation:

(1) $$\chi \left(\%\right)=\frac{A_{110}+A_{200}+A_{020}}{A_{amorphous}+A_{110}+A_{200}+A_{020}}\times 100$$

in which *χ* represents crystallinity of samples; *A*_110_, *A*_200_, and *A*_020_ are the areas under the peaks at 21.4°, 23.7°, and 36.1° respectively; *A_amorphous_* is the area of the peak at 19.8°. The area of all the peaks could be calculated from peak analysis. As shown in Figure 7b, the results from XRD show that a small content of PS could increase the crystallinities of XLPE. But with the increase of PS content, the crystallinities of samples tended to decrease. The increase of crystallinity for a small loading of PS may be attributed to several reasons. PS participates in the crosslinking reaction with LDPE, thus consumption of DCP becomes less for LDPE and more branches of LDPE molecules could attend the formation of lattices, which is in accordance with the results of FTIR. Meanwhile, PS and LDPE are not compatible, and the polymer surface of PS may therefore induce crystallization of LDPE. A similar behavior has been reported in other polymer blends [20].

**Figure 8:**
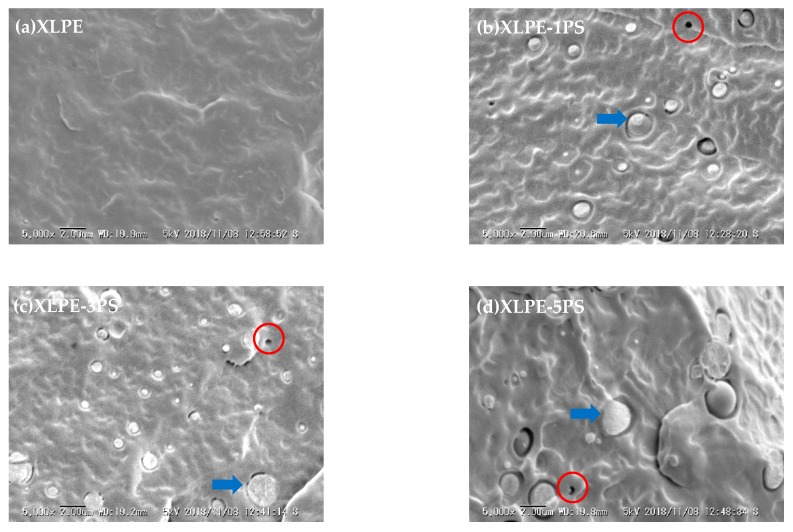
SEM micrographs of cryogenically fractured surfaces for samples. (**a**) XLPE; (**b**) XLPE-1PS; (**c**) XLPE-3PS; (**d**) XLPE-5PS. (Arrow: cross section, circle: cavity).

With the aim of characterizing the interface between XLPE and PS, SEM photographs of cryogenically fractured cross sections of samples are shown in Figure 8. XLPE with PS shows a phase separation and PS dispersed into the matrix of XLPE as spherical particles with a dimension of micrometers, thus forming a typical sea-island structure, which is similar to PP/PS blends reported by Q. Guo et al [21]. When PS is blended with LDPE, interfaces have been introduced between LDPE and PS particles and because of incompatibility, gaps and voids can be generated in the interfacial area. In XLPE with PS particles, however, many PS particles cracked across the section upon the fracture surface (arrow in Figure 8) instead of leaving a cavity (circle in Figure 8), which indicates good binding on the interface between PE and PS particles in spite of phase separation. Combined with the results from FTIR, it could be speculated that the interface of XLPE and PS particles is strengthened by the crosslinking process. Moreover, the average diameter of PS particles was analyzed. The average diameter of XLPE-1PS was 599 nm, XLPE-3PS was 757 nm, and XLPE-5PS was 978 nm. With the increase of PS content, the diameter of these particles tended to increase. Variation in dimensions of PS particles may lead to the change of specific surface area, affecting the consumption of DCP and the formation of the final crosslinking network, which might be the reason why different gel contents and crystallinities are observed. In addition, with the increase of PS content, PS particles become larger and prevent PE spherulites from growing larger, leading to a decrease in crystal size of XLPE with a higher content of PS particles, as shown in Figure 6.

## 4. Discussion

**Figure 9:**
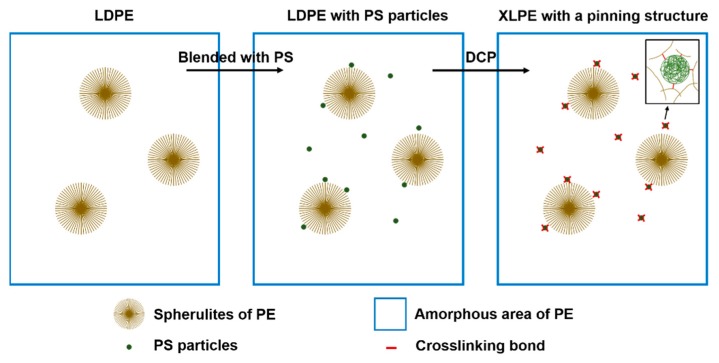
A sketch of XLPE with a polystyrene (PS) pinning structure.

From the results above, it is clear that at first PS disperses into LDPE matrix as spherical particles, and next, by the assistance of DCP, these particles could get access to the crosslinking network. Then during the crystallization, impurities, PS particles, and uncrystallizable polymer are rejected and finally settle in the spherulite boundaries. Hence a pinning structure in XLPE with PS particles come into being, as shown in Figure 9, which is in accordance with SEM photographs of etched surfaces of material samples.

As is known to all, the microstructure of a polymer plays an important role in the DC dielectric performance. Here we discuss the structure–property relationship of XLPE with a PS pinning structure concerning conductivity, space charge, and breakdown strength behaviors.

Basically, carrier transfer in polymers usually occurs at the amorphous region including the spherulite boundary, and the conductivity relies on the concentration of charge carriers, the quantity of charge carriers, and mobility of charge carriers. As presented in Figure 9, PS particles distribute in the amorphous area including the spherulite boundaries and PS has a positive effect on decreasing the carrier mobility [21]. PS particles can impede the transport of charge carriers, thus DC conductivity of XLPE is decreased by the addition of PS particles. With the rise of temperature, the concentration of ions become larger and charge carriers have a higher energy and transport of charge carriers becomes easier. Therefore conductivity of samples tends to increase with the increase of temperature. And in XLPE with PS particles, the crystalline region of XLPE will turn into an amorphous area gradually, decreasing the concentration of PS particles in the amorphous area including the spherulite boundaries. So differences in conductivity between XLPE and XLPE with PS particles tends to decrease with the increase of temperature, but conductivity of XLPE with PS particles is still much lower than that of XLPE at elevated temperatures.

Net space charge in a polymer depends on electrode injection, ionization of impurities, and the charge transfer process, and the trap in XLPE materials is one of the crucial factors [22,23,24]. As shown in Figure 8, a lot of interfaces come into being and benzene rings exist in XLPE with PS particles, both of which can act as traps. Under DC stress larger than 10 kV/mm, injected charge carriers from electrodes could be captured by these interfaces and benzene rings of PS, leading to the accumulation of homocharge. The trap density would increase at a higher PS loading, therefore heavy charge accumulation near the cathode could be observed in the bulk of XLPE-5PS as shown in Figure 2. 

Breakdown phenomenon is a process in which conduction electrons are accelerated by applied DC electric fields and finally develop into an electron avalanche. Factors including material parameters, additives, structure defects, space charge accumulation, and so on, may contribute to the breakdown behavior of materials. From the point of view of structure, on one hand, the higher the crystallinity is, the higher the breakdown strength [20,25]. As shown in Figure 7b, with the increase of PS content, the crystallinity of XLPE tends to decrease, so DC breakdown strength of XLPE with PS particles decreases. On the other hand, investigations carried out by S.N. Kolesov [26] confirm that the discharge channel in spherulitic polymers takes place mostly in the inter-spherulitic space or along the spherulite boundary. During the formation of the discharge channel, introduction of PS particles could absorb the kinetic energy of electrons by means of the benzene ring and thus the existence of PS particles could suppress the formation of a discharge channel. It is also noticed that at 30 °C, DC breakdown strength of XLPE-5PS is lower than that of XLPE and that may attribute to the space charge accumulation. As observed in Figure 2, more charge accumulates in the samples with a higher PS content. At the same time, a lower conductivity under high temperatures could reduce the risk of thermal runway for XLPE with PS particles. As a result, DC breakdown strength of XLPE with PS particles at high temperatures are increased by at least 30%. 

The reason for the enhanced high-temperature DC performances might be ascribed to the addition of PS with a high glass transition temperature (*T*_g_), which means the polymer could maintain the arrangement of molecule chains or segments under a higher temperature. In our investigation, due to the incompatibility of PS and PE, the interface in the heterogeneous structures would form between two phases with separated *T*_g_, i.e., PE (about −70 °C) and PS (about 100 °C). Then the steady crosslinked interface between XLPE and PS particles were further introduced by cross-linking, which pins the inter-spherulitic area in the investigated temperature range and might lead to the enhancement of the energy barrier for charge (electrons or ions) migration compared with ordinary XLPE. Further charge behaviors on the interfaces still remains an open question [8,27,28].

## 5. Conclusions

In this study, a PS pinning XLPE with enhanced DC dielectric properties has been reported, and its high temperature performance has been evaluated. The results show that the introducing of PS can effectively enhance DC breakdown strength and reduce DC conductivity of XLPE at an evaluated temperature up to 90 °C. Further structure study suggests that the PS particles effectively influence the electrical properties of XLPE though the formation of a pinning structure as well as the contained benzene rings. Our finding may provide a novel solution for developing HVDC cable insulation materials.
